# Supplementary material for: Content-rich biological network constructed by mining PubMed abstracts
Source: BMC Bioinformatics. 2004 Oct 8;5:147. doi: 10.1186/1471-2105-5-147 (PMC528731; doi:10.1186/1471-2105-5-147)
Supplement: Additional File 2 — The original results of the above study (non-essential files are deleted to keep the file size under the limit set by BMC bioinformatics). [file 1471-2105-5-147-S2.bz2 › chilibotAdditionalFile2/dip10/227ID7954478E744/html/CTNNB1_CTNNA1.html]

 


 **CTNNB1** and **CTNNA1** 
  
Found 3187 abstracts in PubMed, retrieved 10.  
 

 What does Google say? 
 PDF only 
| .edu only 

---

**Interactive relationship** (e.g. stimulation, inhibition, etc)

**Non-interactive relationship** (e.g. studied together, co-existance, homology, etc.)

- Expression of E cadherin, alpha catenin  [ **CTNNA1** ]   [ **CTNNB1** ] , and beta catenin  [ **CTNNA1** ]   [ **CTNNB1** ]  in the process of lymph node metastasis in oral squamous cell carcinoma.  Ref: 12888830 Br J Cancer, 2003
- TGF beta1 mediated MAP kinase activation also controls Wnt 7a gene expression and Wnt mediated signaling through the intracellular beta catenin  [ **CTNNA1** ]   [ **CTNNB1** ]  TCF pathway, which likely regulates N cadherin expression and subsequent N cadherin mediated cell adhesion complexes during the early steps of MPC chondrogenesis.  Ref: 12893825 J Biol ChemJ Biol Chem, 2003
- In support of this we show that separation of thymocytes from Wnt producing epithelial cells and the thymic microenvironment, triggers beta catenin  [ **CTNNA1** ]   [ **CTNNB1** ]  phosphorylation and degradation in thymocytes.  Ref: 12884861 Eur J Immunol, 2003
- Src activity was increased in QLalpha12 MDCK cells as assessed by Src autophosphorylation, and beta catenin  [ **CTNNA1** ]   [ **CTNNB1** ]  tyrosine phosphorylation was also increased, although there was no detectable increase in Rho activity.  Ref: 12890651 Am J Physiol Cell PhysiolAm J Physiol Cell Physiol, 2003
- Thus, sustained exposure to Wnt in the context of an intact stromal microenvironment is necessary for stabilization of beta catenin  [ **CTNNA1** ]   [ **CTNNB1** ]  mediated signaling in thymocytes.  Ref: 12884861 Eur J Immunol, 2003
- Western blotting was used to determine polypeptide size and expression of E cadherin and beta catenin  [ **CTNNA1** ]   [ **CTNNB1** ]  in Wilms s tumours compared with normal kidney.  Ref: 12890743 Mol Pathol, 2003
- Low to high gamma catenin  [ **CTNNA1** ]   [ **CTNNB1** ]  and ezrin expression was seen in cytoplasm of blastemal and epithelial cells of primary and metastatic tumours.  Ref: 12890743 Mol Pathol, 2003
- beta catenin  [ **CTNNA1** ]   [ **CTNNB1** ]  is one of the essential components of this specification.  Ref: 12885771 J Biol ChemJ Biol Chem, 2003
- p120 catenin  [ **CTNNA1** ]   [ **CTNNB1** ]  p120 was originally identified as a tyrosine kinase substrate, and subsequently shown to regulate cadherin mediated cell cell adhesion.  Ref: 12885254 Biochemistry, 2003
- Here we show that beta catenin  [ **CTNNA1** ]   [ **CTNNB1** ]  signaling is required for the formation of the distal, but not the proximal, airways.  Ref: 12885771 J Biol ChemJ Biol Chem, 2003
- CONCLUSIONS Compared with primary tumours, metastatic tumours showed lower expression of E cadherin and gamma catenin  [ **CTNNA1** ]   [ **CTNNB1** ] , with nuclear staining for beta catenin  [ **CTNNA1** ]   [ **CTNNB1** ] .  Ref: 12890743 Mol Pathol, 2003
- We have studied the effects of the organophosphorous pesticide malathion on cell viability, actin cytoskeleton, cell adhesion complex E cadherin beta catenin  [ **CTNNA1** ]   [ **CTNNB1** ] , and Rho and Rac1 GTPases from the human mammary carcinoma cell line MCF 7.  Ref: 12888906 Int J Oncol, 2003
- Primary and metastatic tumours showed moderate to high beta catenin  [ **CTNNA1** ]   [ **CTNNB1** ]  expression in blastemal and epithelial cells, with predominantly membranous and cytoplasmic staining.  Ref: 12890743 Mol Pathol, 2003
- METHODS Immunohistochemistry was used to determine the expression and cellular distribution of E cadherin, beta catenin  [ **CTNNA1** ]   [ **CTNNB1** ] , gamma catenin  [ **CTNNA1** ]   [ **CTNNB1** ] , and ezrin in primary and metastatic Wilms s tumours.  Ref: 12890743 Mol Pathol, 2003
- These data suggest that evaluation of the immunohistochemical expression of E cadherin, alpha catenin  [ **CTNNA1** ]   [ **CTNNB1** ]  and beta catenin  [ **CTNNA1** ]   [ **CTNNB1** ]  is extremely valuable for the diagnosis of metastatic occurrence.British Journal of Cancer 2003 8 57 563.  Ref: 12888830 Br J Cancer, 2003
- Higher amounts of 92 kDa beta catenin  [ **CTNNA1** ]   [ **CTNNB1** ]  were detected in tumours than in normal kidney.  Ref: 12890743 Mol Pathol, 2003
- In the metastatic process, reduction in cell to cell adhesion including E cadherin catenin  [ **CTNNA1** ]   [ **CTNNB1** ]  cell adhesion complex is an essential step.  Ref: 12888830 Br J Cancer, 2003
- Significantly greater reduction in expression levels of E cadherin, alpha catenin  [ **CTNNA1** ]   [ **CTNNB1** ]  and beta catenin  [ **CTNNA1** ]   [ **CTNNB1** ]  was found in the metastatic group n=64 compared to the nonmetastatic group n=95 P=0.00.  Ref: 12888830 Br J Cancer, 2003
- Expression of non destructible beta catenin  [ **CTNNA1** ]   [ **CTNNB1** ]  mutants increased survivin expression and protected against ultraviolet B induced apoptosis.Interpretation Stimulation of survivin expression by TCF beta catenin  [ **CTNNA1** ]   [ **CTNNB1** ]  might impose a stem cell like phenotype to colonic crypt epithelium coupling enhanced cell proliferation with resistance to apoptosis, and contribute to the molecular pathogenesis of colorectal cancer.  Ref: 12885482 Lancet, 2003
- beta catenin  [ **CTNNA1** ]   [ **CTNNB1** ]  is required for specification of proximal distal cell fate during lung morphogenesis.  Ref: 12885771 J Biol ChemJ Biol Chem, 2003
- Accumulating evidence has highlighted the importance of beta catenin  [ **CTNNA1** ]   [ **CTNNB1** ]  mediated activation of T cell factor TCF lymphoid enhancer factor LEF transcription during thymocyte development.  Ref: 12884861 Eur J Immunol, 2003
- Changes in apoptosis were monitored in cell lines engineered to express stabilising mutations in beta catenin  [ **CTNNA1** ]   [ **CTNNB1** ] .Findings TCF beta catenin  [ **CTNNA1** ]   [ **CTNNB1** ]  stimulated a six fold to 12 fold increased expression of the survivin gene in colorectal cancer cells.  Ref: 12885482 Lancet, 2003
- We investigated immunohistochemical expression of E cadherin, alpha catenin  [ **CTNNA1** ]   [ **CTNNB1** ]  and beta catenin  [ **CTNNA1** ]   [ **CTNNB1** ]  in 159 tissue samples from patients with oral squamous cell carcinoma and examined the correlation between their expressions and the presence of regional lymph node metastasis.  Ref: 12888830 Br J Cancer, 2003
- When the gene for beta catenin  [ **CTNNA1** ]   [ **CTNNB1** ]  was conditionally excised in epithelial cells of the developing mouse lung prior to E14.5, the proximal lung tubules grew and differentiated appropriately.  Ref: 12885771 J Biol ChemJ Biol Chem, 2003
- AIMS To evaluate E cadherin, beta catenin  [ **CTNNA1** ]   [ **CTNNB1** ] , gamma catenin  [ **CTNNA1** ]   [ **CTNNB1** ] , and ezrin expression and its relation to histopathological features of primary and metastatic Wilms s tumours.  Ref: 12890743 Mol Pathol, 2003
- BACKGROUND The E cadherin catenin  [ **CTNNA1** ]   [ **CTNNB1** ]  adhesion complex is crucial for intercellular adhesiveness and maintenance of tissue architecture.  Ref: 12890743 Mol Pathol, 2003
- beta Catenin  [ **CTNNA1** ]   [ **CTNNB1** ]  mutations in liver tumors induced by 2 amino dimethylimidazo f quinoline in CDF 1 mice.  Ref: 12893427 Cancer Lett, 2003
- Adhesion Associated and PKC Modulated Changes in Serine Threonine Phosphorylation of p120 Catenin  [ **CTNNA1** ]   [ **CTNNB1** ] .  Ref: 12885254 Biochemistry, 2003
- These changes were related to a decrease in the expression of the adhesion molecules, E cadherin and beta catenin  [ **CTNNA1** ]   [ **CTNNB1** ] , and to the distribution and reactivity of actin microfilaments to TRITC phalloidin.  Ref: 12888906 Int J Oncol, 2003
- Background Colorectal cancer is thought to originate in the expansion of colonic crypt cells as a result of aberrant gene expression caused by transcription factors of the T cell factor TCF beta catenin  [ **CTNNA1** ]   [ **CTNNB1** ]  family.  Ref: 12885482 Lancet, 2003
- In this study, we screened liver tumors induced by 2 amino dimethylimidazo f quinoline MeIQ in CDF 1 mice for beta catenin  [ **CTNNA1** ]   [ **CTNNB1** ]  and APC mutations and other genetic alterations shown to occur in human hepatocellular carcinomas HCC, including mutations in the p53 and H ras genes, c myc amplification and E cadherin promoter methylation.  Ref: 12893427 Cancer Lett, 2003
- These data indicate that except for the occasional disruption of the Wnt pathway through beta catenin  [ **CTNNA1** ]   [ **CTNNB1** ]  mutations, the genetic pathways involved in the development of HCC differ significantly between human liver cancer and tumors induced in mice by MeIQ, but do not rule out the possibility that heterocyclic amines constitute a carcinogenic risk factor in humans.  Ref: 12893427 Cancer Lett, 2003
- SSCP followed by direct DNA sequencing revealed mutations in exon 2 of the beta catenin  [ **CTNNA1** ]   [ **CTNNB1** ]  gene in 2 of 16 liver tumors 12.5%.  Ref: 12893427 Cancer Lett, 2003
- Differential expression of E cadherin and beta catenin  [ **CTNNA1** ]   [ **CTNNB1** ]  in primary and metastatic Wilms s tumours.  Ref: 12890743 Mol Pathol, 2003
